# Prolactin-induced mouse mammary carcinomas model estrogen resistant luminal breast cancer

**DOI:** 10.1186/bcr2819

**Published:** 2011-01-28

**Authors:** Lisa M Arendt, Debra E Rugowski, Tara A Grafwallner-Huseth, Maria Jose Garcia-Barchino, Hallgeir Rui, Linda A Schuler

**Affiliations:** 1 Department of Comparative Biosciences, University of Wisconsin--Madison, 2015 Linden Dr., Madison, WI 53706, USA; 2 Department of Cancer Biology, Kimmel Cancer Center, Thomas Jefferson University, 233 South 10th St, 332 BLSB, Philadelphia, PA 19107, USA; 3 Department of Anatomy & Cellular Biology, Sackler School, Tufts University School of Medicine, 136 Harrison Ave, Boston, MA 02111, USA; 4 Genetics Department, Navarra University, Irunlarrea St. 1, Pamplona, 31008, Spain

## Abstract

**Introduction:**

Tumors that express estrogen receptor alpha (ERα+) comprise 75% of breast cancers in women. While treatments directed against this receptor have successfully lowered mortality rates, many primary tumors initially or later exhibit resistance. The paucity of murine models of this "luminal" tumor subtype has hindered studies of factors that promote their pathogenesis and modulate responsiveness to estrogen-directed therapeutics. Since epidemiologic studies closely link prolactin and the development of ERα+ tumors in women, we examined characteristics of the aggressive ERα+ and ERα- carcinomas which develop in response to mammary prolactin in a murine transgenic model (neu-related lipocalin- prolactin (NRL-PRL)). To evaluate their relationship to clinical tumors, we determined phenotypic relationships among these carcinomas, other murine models of breast cancer, and features of luminal tumors in women.

**Methods:**

We examined a panel of prolactin-induced tumors for characteristics relevant to clinical tumors: histotype, ERα/progesterone receptor (PR) expression and estrogen responsiveness, Activating Protein 1 (AP-1) components, and phosphorylation of signal transducer and activator of transcription 5 (Stat5), extracellular signal regulated kinase (ERK) 1/2 and AKT. We compared levels of transcripts in the ERα-associated "luminal" signature that defines this subtype of tumors in women and transcripts enriched in various mammary epithelial lineages to other well-studied genetically modified murine models of breast cancer. Finally, we used microarray analyses to compare prolactin-induced ERα+ and ERα- tumors, and examined responsiveness to estrogen and the anti-estrogen, Faslodex, *in vivo*.

**Results:**

Prolactin-induced carcinomas were markedly diverse with respect to histotype, ERα/PR expression, and activated signaling cascades. They constituted a heterogeneous, but distinct group of murine mammary tumors, with molecular features of the luminal subtype of human breast cancer. In contrast to morphologically normal and hyperplastic structures in NRL-PRL females, carcinomas were insensitive to ERα-mediated signals. These tumors were distinct from mouse mammary tumor virus (MMTV)-neu tumors, and contained elevated transcripts for factors associated with luminal/alveolar expansion and differentiation, suggesting that they arose from physiologic targets of prolactin. These features were shared by ERα+ and ERα- tumors, suggesting a common origin, although the former exhibited transcript profiles reflecting greater differentiation.

**Conclusions:**

Our studies demonstrate that prolactin can promote diverse carcinomas in mice, many of which resemble luminal breast cancers, providing a novel experimental model to examine the pathogenesis, progression and treatment responsiveness of this tumor subtype.

## Introduction

The hormone, prolactin (PRL), is critical for the development and functional differentiation of the mammary gland [1]. Although its physiologic importance suggests a role in breast carcinogenesis, such activity has been controversial. However, accumulating evidence from a variety of sources now supports a link between this hormone and breast disease. Large prospective epidemiologic studies have correlated circulating levels of PRL with an increased risk of particularly estrogen receptor positive (ERα+) invasive tumors [2]. Although the relationship between circulating PRL and patient survival has been examined only in smaller studies, higher levels have been associated with tumor aggression, higher risk of metastasis and poor long term survival (reviewed in [2,3]). Moreover, the pituitary is not the only source of PRL to the breast. PRL is also expressed within the mammary gland, particularly in humans, permitting autocrine/paracrine actions [4-6]. Recent studies have begun to link genetic variations in the genes for PRL and the prolactin receptor (PRLR) and breast cancer [7-9]. Furthermore, in addition to PRL itself, human growth hormone is also a potent agonist at the PRLR; thus PRLR-transduced signals also may mediate some signals of this other oncogenic hormone [10,11]. Finally, many primary breast tumors, both ERα+ and ERα-, express the PRLR, pointing to its potential utility as a therapeutic target and prognostic indicator [4,12,13].

Although epidemiological data support a role for PRL in the development and progression of breast cancer, relatively little is known about its contributions to this disease. In order to investigate the pathogenic actions of PRL, we have developed a transgenic mouse model (NRL-PRL), which mimics the high mammary PRL synthesis observed in women. In this model, the PRL- and estrogen- insensitive NRL promoter drives expression of the rat PRL transgene in mammary epithelia, exposing the gland to locally elevated PRL [14,15]. Nonparous NRL-PRL females develop mammary pathology that exhibits many features of human disease, including early lesions (hyperplasias and intraepithelial neoplasias, similar to ductal carcinoma in situ in women), and eventually, ERα+ and ERα- carcinomas. These tumors are locally aggressive, and metastases to local lymph nodes and lungs are occasionally observed [16]. The development of tumors in NRL-PRL females is not dependent on postpubertal ovarian steroids, but is accelerated by supplemental estrogen [17].

Expression of ERα has emerged as the foremost prognostic and therapeutic indicator in primary clinical breast tumors. However, ERα+ cancers are themselves very diverse, and many are not susceptible to treatments directed at this pathway. Indeed, 25% of women treated with tamoxifen will succumb to breast cancer [18]. Large scale transcript profiling of clinical tumors has begun to reveal the basis of ERα+ cancer diversity, as well as that of other tumor subtypes defined by pathologic markers (for review, [19]). ERα+ tumors share the "luminal" transcript signature, which includes the ERα itself and several other genes linked to ERα expression, as well as defining features such as luminal cytokeratins [19,20]. Efforts to resolve luminal tumors into subtypes that predict therapeutic sensitivity have demonstrated elevated expression of proliferation-related genes in patients at higher risk for relapse, which are useful clinically [21-23]. However, these studies have not revealed vulnerabilities that can be therapeutically targeted.

Mouse models have proven to be useful in elucidating the origins of tumor subtypes, and have revealed complex etiologies and relationships to mammary epithelial lineages [24,25]. However, the paucity of murine models of ERα+ tumors has hindered studies of the factors that give rise to this prevalent tumor subtype and modulate responsiveness to estrogen-directed therapeutics. In light of the epidemiologic evidence linking PRL exposure to the development of ERα+ cancers in women, we investigated the phenotype of the mammary carcinomas that develop in NRL-PRL females, with regard to features defining clinical luminal tumors, including expression of the "ERα-associated signature", estrogen sensitivity, and activated signaling pathways. Our studies demonstrate that PRL can promote diverse tumor phenotypes, many of which display molecular features of luminal breast cancers, providing insight into the pathogenesis of this cancer subtype.

## Materials and methods

### Reagents

5-bromo-2-deoxyuridine (BrdU) was obtained from Sigma Chemical Co. (St. Louis, MO, USA), and 17β-estradiol (E2) was purchased from Steraloids, Inc. (Newport, RI, USA. The following antibodies were used for immunohistochemical analyses: BrdU (MAS-250) from Accurate Scientific (Westbury, NY, USA), proliferating cell nuclear antigen (PCNA; PC 10) and progesterone receptor (PR; A0098) from DAKO Cytomation (Carpinteria, CA, USA), c-Fos (SC-52) and estrogen receptor alpha (ERα; SC-542) from Santa Cruz Biotechnology, Inc. (Santa Cruz, CA, USA), pERK 1/2 (Thr202/Tyr204; 9102), pAkt (S473; 3787), and c-Jun (9162) from Cell Signaling Technology (Beverly, MA, USA), pStat5 (AX1) from Advantex BioReagents, LLP (El Paso, TX, USA).

### Mouse models

NRL-PRL mice (line 1655-8, TgN(Nrl-Prl)24EPS) were generated and maintained in the FVB/N strain background as described [14]. Mice were housed and handled in accordance with the Guide for Care and Use of Laboratory Animals in AAALAC-accredited facilities. All procedures were approved by the University of Wisconsin-Madison Animal Care and Use Committee. p53^+/- ^heterozygotes [26] that were backcrossed more than 10 generations into the FVB/N strain [27] were used to generate p53^-/- ^donors. p53^-/- ^mammary epithelial cells (MECs) were transplanted into FVB/N nontransgenic female recipients, and the resulting tumors harvested (manuscript in preparation, O'Leary and Schuler). Mammary tumors from other genetically modified murine models (FVB/N) were generously provided by Drs. Kim and Alexander (University of Wisconsin) (MMTV-neu [28], and Drs. Green and Zi-Yao Liu (NIH-NCI) (C3(1)-SV40 Tag tumors [29].

### Histological examination of mammary tissue

For some studies, mice were injected with 200 mg/kg body weight BrdU 1 h prior to sacrifice to label cells undergoing DNA synthesis. For immunohistochemistry (IHC), deparaffinized slides were exposed to 0.5% H_2_O_2 _in methanol to block endogenous peroxidase activity, boiled for 15 minutes in 0.1 M citrate (pH = 6.0) or 0.1 M Tris (pH = 9.0) buffer for antigen retrieval, then blocked, and incubated with the primary antibody overnight (for individual antibodies, see Table S1 in Additional File 1). Slides were incubated with the secondary antibody (BioGenex, San Ramon, CA, USA), followed by peroxidase strepavidin and 3,3' diaminobenzidine, and counterstained with hematoxylin. Cells were labeled for PCNA using Vector M.O.M. Basic kit (BMK-2202; Vector Laboratories, Burlingame, CA, USA). pStat 5 was detected as described [30]. Serial sections of tumors were examined for the different endpoints. For all IHC, control sections were stained in parallel (negative controls: omission of primary antibody, irrelevant antibody; positive controls, appropriate for the antibody) as described [14]. To determine labeling indices, 1,000 total cells in at least five randomly chosen microscopic fields in divergent regions of each carcinoma were counted by an investigator blind to the experimental status.

### Microarray experiments

In a preliminary study to compare the tumors that develop in NRL-PRL to other mouse models of mammary cancer, cDNA prepared from tumors was hybridized to Agilent Mouse Oligo Microarrays (Agilent Technologies Inc., Santa Clara, CA, USA) using total RNA from equal numbers of C57Bl6/J and 129 male and female pups as the reference sample, as previously described [31]. Data were processed and hierarchical clustering performed, as described [31].

To compare NRL-PRL tumors identified as ERα positive or negative by IHC, total RNA was purified using RNeasy Midi Kits (Qiagen Inc., Valencia, CA, USA) according to the manufacturer's instructions. RNA integrity was assessed using the RNA 6000 Nano Assay and Agilent 2100 Bioanalyzer (Agilent Technologies Inc.). Total RNA (10 μg) was reverse transcribed and labeled with Cy5 using the Array 50 kit (Genisphere Inc., Hatfield, PA, USA). MECs were isolated as described [32] from 12-week-old nontransgenic nonparous FVB/N females, for common reference RNA. Reference RNA pooled from 15 mice was reverse transcribed and labeled with Cy3. Tumor and reference samples were co-hybridized overnight to Agilent Mouse cDNA Microarrays (G4104A, 8,614 features), washed, and scanned using Agilent's dual-laser Microarray Scanner (G2565BA) with Feature Extraction Software.

Genes with Cy3 and Cy5 intensity values higher than 100 were considered as expressed genes and were normalized with lowess smoother followed by log base 2 transformation. Values that changed at least 1.5-fold in either direction from the gene's median value were included in the class comparison. Genes differentially expressed between ERα positive and negative classes were determined with a significance threshold of univariate tests at *P *< 0.01. Average linkage hierarchical clustering on both the genes and samples was performed using the gene list generated by class comparison. Analyses were performed using BRB ArrayTools (version 3.6.0) developed by Dr. Richard Simon and Amy Peng Lam (Biometric Research Branch, National Cancer Institute). These data have been deposited in the public database, ArrayExpress (E-MEXP-3013).

### Real-time PCR

RNA from tumors and MECs was examined using quantitative real time PCR (qRT-PCR). cDNA was synthesized from 1 μg RNA using random hexamers (Amersham Biosciences, Piscataway, NJ, USA) and MMLV Reverse Transcriptase (Promega, Madison, WI, USA), and qRT-PCR performed as described [17]. Negative (no cDNA) and positive controls were included with each plate. Specific primer sequences are listed in Table S2 in Additional File 1. Results were calculated using the comparative C_T _method and normalized to 18S RNA, and data were analyzed for statistical significance as described in the figure legends.

### Tumor sensitivity to ovarian steroids

To examine the responsiveness of tumors to circulating ovarian steroids, tumors were allowed to develop until they reached 1.5 cm in diameter. About one-third of the tumor mass was then removed, concomitant with ovx or sham surgery, or ovx and subcutaneous treatment with 0.1 mg pellets of 17β-estradiol (E2; Innovative Research of America, Sarasota, FL, USA), which result in plasma levels of 50 to 75 pg/ml [33]. Tumors were measured twice weekly with calipers, and tumor volume was calculated (the largest diameter x (the smallest diameter)^2 ^× 0.4). Mice were humanely euthanized and tissue collected when the tumors reached 1.5 cm in diameter. Histological comparisons of the tumor biopsies and end stage tumors were carried out as above.

For some experiments, 1 mm^3 ^NRL-PRL tumor fragments were transplanted bilaterally into fourth inguinal mammary glands of 12-week-old nontransgenic syngeneic FVB/N hosts. After the tumors grew to 0.75 cm in diameter, recipient females were treated with ovx, sham surgery, or sham surgery and weekly subcutaneous injections of 5 mg Faslodex (ICI 182,780; AstraZeneca, Wilmington, DE, USA). Tumors were measured and collected as described above.

### Statistical analyses

Statistical analyses were performed using Prism v.4.03 (GraphPad Software, Inc., San Diego, CA, USA). Differences were considered significant at *P *< 0.05.

## Results and discussion

### Locally elevated PRL leads to histologically diverse ERα positive and ERα negative carcinomas

**Figure 1:**
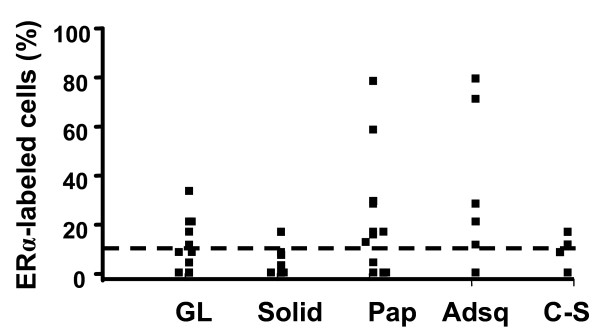
**Mammary carcinomas that develop in NRL-PRL females exhibit diverse histotypes, and express varying levels of ERα**. The proportion of cells containing immuno-detectable ERα was quantitated as described in Materials and methods, and shown related to tumor histotype. Each box represents a single tumor that arose in a different NRL-PRL nonparous female. The dashed line marks 10% ERα+ cells, a common clinically used threshold. (Tumor histotypes: GL, glandular; Pap, papillar; Adsq, adenosquamous; C-S, carcinosarcoma).

NRL-PRL nonparous females develop diverse invasive mammary carcinomas with a long latency [14]. In order to examine the breadth of tumor phenotypes promoted by this prolonged PRL exposure, we examined a panel of 39 of these tumors (mean latency +/- standard deviation (S.D.), 18.2+/-3.6 months). The majority were adenocarcinomas of several histotypes; carcinosarcomas constituted a minor population (Figure 1, Table 1). These carcinomas varied widely in the proportion of cells that expressed detectable ERα (0 to 79%). Nineteen of the 39 tumors examined exhibited greater than 10% ERα+ cells, a common clinically used threshold. The majority did not express detectable progesterone receptor (PR); only four tumors demonstrated PR immunostaining in more than 5% of the tumor cells. The morphological heterogeneity suggests cooperation between PRL and other oncogenic factors during the long period of development of these tumors, similar to that which may occur in women.

**Table 1:** Characteristics of PRL-induced carcinomas by histotype

	Glandular	Solid	Papillary	Adenosquamous	Carcinosarcoma
N	10	3	10	3	4
ERα^1^	10.0 ± 3.6 ^a^	4.4 ± 2.9 ^b^	21.1 ± 8.1	32.2 ± 10.2 ^a,b^	8.2 ± 3.0
PR^1^	1.5 ± 1.0	0.3 ± 0.2	13.8 ± 6.3	2.4 ± 1.4	0
pStat5^1^	49.0 ± 8.13^a,b^	66.6 ± 12.2 ^c,d,e^	29.4 ± 18.7 ^c^	23.9 ± 5.25 ^a,d^	5.7 ± 1.9 ^b,e^
c-Fos^1^	18.3 ± 2.8 ^a^	15.0 ± 2.5^b,c^	17.6 ± 3.7 ^d^	30.5 ± 4.4 ^a,b^	25.2 ± 3.2 ^c,d^
S phase^1^	15.6 ± 1.7	15.0 ± 2.5	18.0 ± 2.0	14.7 ± 1.3	15.5 ± 2.2
Apoptotic^1^	2.1 ± 0.3	3.6 ± 1.1	2.2 ± 0.3	1.9 ± 0.4	1.1 ± 0.2
pERK1/2^2^	70% strong, 10% weak stromal; 30% epithelial	100% strong stromal	90% strong stromal; 10% epithelial	100% strong stromal	100% strong stromal & epithelial
pAKT^2^	50% epithelial; 20% stromal; 30% negative	67% epithelial; 33% negative	70% epithelial; 10% stromal; 20% negative	33% epithelial & stromal; 67% negative	75% epithelial; 25% negative

^1^% tumor epithelial cells that are positive, mean ± s.e.m (see Materials and methods). Same lower case letters indicate significant differences between histotypes (*P *< 0.05; Students *t*-test).

^2^% of tumors of each histotype that displayed strong staining in different locations (stroma, epithelium, or both) as indicated.

ERα, estrogen receptor alpha; ERK, extracellular signal regulated kinase; PR, progesterone receptor; PRL, prolactin; Stat5, signal transducer and activator of transcription 5.

### PRL-induced tumors display inverse Stat5 and AP-1 activation and elevated epithelial pAKT

**Figure 2:**
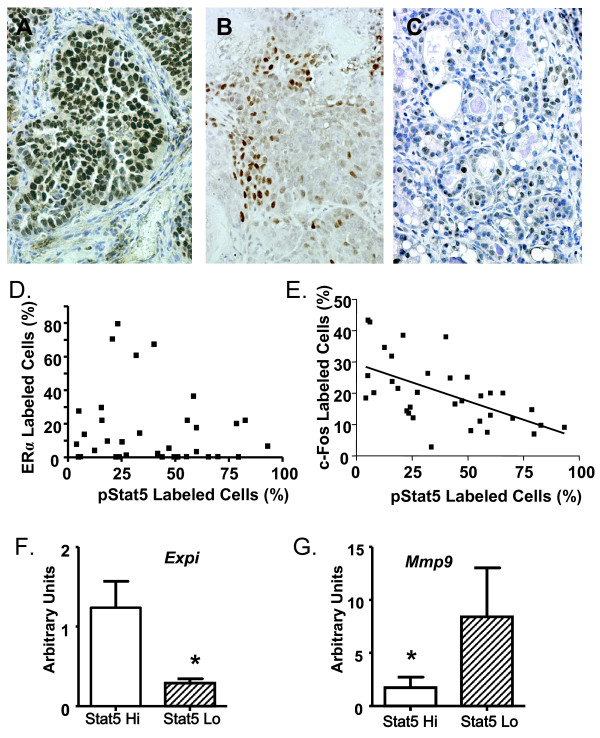
**PRL-induced carcinomas demonstrate an inverse relationship between nuclear phosphorylated Stat 5 (pStat 5) and AP-1 proteins**. **(a)** Nuclear pStat 5 staining in a glandular adenocarcinoma. **(b) **c-Fos expression in a glandular adenocarcinoma. **(c)** c-Jun staining in a glandular adenocarcinoma. **(d)** The proportion of tumor cells that express ERα and nuclear pStat5 is not correlated. **(e)** Carcinomas exhibit a significantly negative correlation between the proportion of cells that contain nuclear pStat 5 and c-Fos (*P *< 0.0001, R = -0.63). ERα, pStat 5, c-Fos and c-Jun expression were evaluated and quantitated as described in Materials and Methods. Each box represents a single carcinoma that arose in a different NRL-PRL nonparous female. Correlations were determined using Spearman's non-parametric test. **(f)***Expi *mRNA is significantly increased in the adenocarcinomas expressing highest pStat5, compared to the lowest 5 (mean ± s.e.m., *N *= 5; *, *P *= 0.003). **(g)***Mmp9 *mRNA is significantly decreased in the adenocarcinomas expressing highest pStat5, compared to the lowest 5 (mean ± s.e.m., *N *= 5; *, *P *= 0.03). (f, g) Transcript levels were measured using qRT-PCR as described in Materials and Methods. Original magnification: a, b, c, 400x.

PRL can activate multiple signaling cascades, which may play distinct roles in its physiologic and pathogenic actions in the mammary gland. In order to determine the relationship of these signals to tumor characteristics, we examined the activation of these pathways in the panel of PRL-induced tumors by IHC. Signal transducer and activator of transcription 5 (Stat5) is one of the best characterized mediators of PRL activity, and is critical for PRL-induced alveolar development during pregnancy (for review, [1,34]). The tumors that developed in NRL-PRL females contained variable proportions of cells with nuclear pStat5 (range: 4.2 to 93.3%), which was not related to ERα levels (Figure 2a, d, *P *> 0.05). Although many glandular and solid tumors exhibited relatively high proportions of cells containing nuclear pStat5, evidence for activation of this pathway varied substantially even among these histotypes. Many adenocarcinomas demonstrated low nuclear pStat5, as did all of the poorly differentiated carcinosarcomas (Table 1).

PRL can also initiate other signals, including several MAP kinases, which are elevated in mammary lysates of NRL-PRL females [17], and are particularly strongly activated in some breast cancer cells lines [35]. PRL-activated MAP kinases can increase synthesis and phosphorylation of multiple Activating Protein-1 (AP-1) components, activating the AP-1 transcriptional enhancer [36]. AP-1 target genes have been shown to enhance cellular proliferation, survival, and invasion (for review, [37-39]). Both c-Fos and c-Jun were variably expressed in PRL-induced tumors (Figure 2b, c, e*)*. Their expression was directly related to each other (data not shown, *P *= 0.005, R = 0.83). Although adenosquamous carcinomas and carcinosarcomas had the highest levels of c-Fos expression, a large subset of other histotypes also displayed significant staining (Table 1). Interestingly, nuclear pStat 5 levels were negatively correlated with both c-Fos (Figure 2e; *P *< 0.0001, R = -0.63) and c-Jun (data not shown; *P *= 0.0058, R = -0.82). The inverse relationship of these signals in primary NRL-PRL tumors is consistent with our previous observation of PRL signals to Stat5 and AP-1 in several breast cancer cell lines [35].

The higher level of nuclear pStat5 in well-differentiated PRL-induced carcinoma histotypes resembles the association of pStat5 with features of more differentiated clinical breast tumors (for review, [34]). In order to examine the association of this pathway with molecular phenotype in adenocarcinomas, we examined levels of two transcripts associated with distinct mammary behaviors. The 5 PRL-induced adenocarcinomas with highest nuclear pStat5 (70 to 93%) and low c-Fos had significantly elevated transcripts for the proteinase inhibitor, Expi, compared to those adenocarcinomas with lowest pStat5 (4 to 10%, Figure 2f, *P *= 0.003). Expi expression increases in early pregnancy [40], and is reduced in PRLR^-/- ^mammary glands [41]. In contrast, the adenocarcinomas with lowest nuclear pStat5 contained elevated mRNA for the metalloproteinase, *Mmp9 *(Figure 2g; *P *= 0.03), which in women is associated with high tumor grade, metastasis, and reduced survival [42], consistent with a more aggressive phenotype.

**Figure 3:**
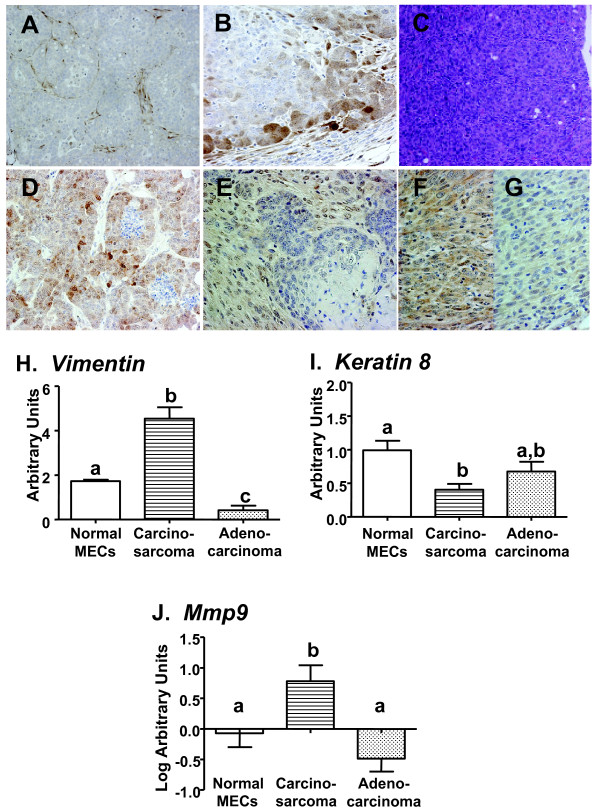
**Adenocarcinomas exhibit activated ERK1/2 in stroma and AKT in epithelia; carcinosarcomas display strong epithelial pERK1/2**. **(a)** pERK1/2 localized in stromal cells of differentiated glandular adenocarcinomas. **(b)** Mammary carcinosarcomas demonstrated nuclear and cytoplasmic staining for pERK1/2. **(c)** Carcinosarcoma from gland of NRL-PRL female (hematoxylin and eosin stain). **(d)** Glandular adenocarcinoma from NRL-PRL female demonstrating nuclear and cytoplasmic staining for pAKT. **(e)** pAKT localized primarily in the stroma of a squamous adenocarcinoma. **(f, g)** Carcinosarcomas demonstrated variable levels of pAKT expression. **(h)** Carcinosarcomas demonstrated significantly higher levels of vimentin mRNA compared to either morphologically normal MECs or more differentiated adenocarcinomas from NRL-PRL females. **(i)** Carcinosarcomas had similar levels of keratin 8 transcripts compared to adenocarcinomas. **(j)***Mmp9 *mRNA was significantly increased in carcinosarcomas. Transcript levels were measured using qRT-PCR as described in Materials and Methods and represented as mean ± s.e.m. (MECs, *N *= 3 samples, each containing RNA pooled from 5 mice; carcinosarcomas, *N *= 4; adenocarcinomas with high pStat5, *N *= 5). Letters denote significant differences using ANOVA, followed by Newman-Keuls post test (*P *< 0.05). Original magnification: a, c, 200x; b, d, e, f, g, 400x.

Elevation of phosphorylated extracellular signal regulated kinases 1/2 (pERK1/2) is found in some clinical tumors in women [43], and is increased in lysates of NRL-PRL mammary glands [17]. The location of pERK1/2 varied substantially among PRL-induced tumors. The majority of the adenocarcinomas (68%) demonstrated strong stromal staining with rare nuclear staining in epithelial cells (Table 1, Figure 3a). In contrast, all of the carcinosarcomas in the panel displayed both strong epithelial nuclear and cytoplasmic labeling (Figure 3b, c). This histotype had significantly higher levels of *Vim *(Figure 3h), but similar levels of *Krt*8 transcripts compared to the adenocarcinomas (Figure 3i). The spindle shaped cell morphology, loss of cell polarity, and expression of *Vim *mRNA within these tumors suggest the epithelial to mesenchymal transition (EMT, for review, [44,45]). Significantly enhanced levels of *Mmp9 *transcripts were also detected in these tumors (Figure 3j).

The PI3K/AKT pathway also has been associated with proliferation, survival, and EMT in human breast tumors, as well as resistance to endocrine therapy [46-48]. pAKT is also elevated in mammary glands of NRL-PRL females [17]. The majority of the carcinomas in this panel displayed strong pAKT labeling in both the nuclei and cytoplasm of tumor epithelium (Table 1, Figure 3d). Other tumors exhibited pAKT labeled cells in both the stroma and epithelium, and a minority in the stromal compartment alone (Figure 3e). Unlike pERK1/2, pAKT expression was not correlated with a specific histotype; carcinosarcomas demonstrated variable expression levels of nuclear and cytoplasmic pAKT (Figure 3f, g).

Together, these observations suggest that a broad spectrum of signals may contribute to progression of these carcinomas. Nuclear pStat5 is elevated in hyperplastic lesions in NRL-PRL glands [17], and early ablation of Jak2 protects against PRL-induced tumors [49], consistent with an important role for the Jak2-Stat5 pathway in their genesis. However, the data presented here indicate that high pStat5 persists in only a minor subset of established PRL-induced carcinomas, suggesting that other signals drive progression of most of these tumors. Elevated activities of AP-1 [50,51], AKT [52] and MAP kinases, including ERK1/2 [43], have been implicated in resistance to conventional chemotherapies and anti-estrogens. PRL can activate these signals alone, as well as potently cooperate with growth factors to enhance ERK1/2 and AKT activation *in vitro *and *in vivo *[53-55]. In light of the variable levels of *Prlr *transcripts in these tumors (see below), it is clear that PRL itself may play different roles in ongoing activation of these pathways as they influence lesion progression, tumor phenotype, and treatment sensitivity.

### PRL-induced carcinomas share molecular features of luminal tumors in women

To evaluate shared characteristics of PRL-induced carcinomas, in a preliminary study we examined the transcript profiles of six NRL-PRL carcinomas, including three each of the most prevalent histotypes (glandular and papillary), expressing variable levels of ERα (three positive, three negative by IHC, not associated with histotype). Unsupervised hierarchical clustering based on an intrinsic gene list developed for murine models [31] showed that the NRL-PRL tumors formed a distinct subgroup neighboring MMTV-neu tumors.

To compare the phenotype of NRL-PRL tumors more closely to other well-studied genetically modified murine models of breast cancer subtypes in the FVB/N strain background, we compared levels of transcripts for ERα-associated genes in the "luminal" signature [20] and those enriched in various mammary epithelial lineages. MMTV-neu tumors model clinical HER2+ tumors. Like both human HER2+ tumors [25] and other murine tumors that develop in response to MMTV-driven oncogenes [31], MMTV-neu tumors display some characteristics of the "luminal" tumor subtype in women. Tumors in this extensively studied mouse model do not express ERα or FoxA1, a transcription factor which co-regulates many targets [56,57]. However, they do express other ERα-associated genes, including Xbp1 and Gata 3 [58-60]. In contrast, tumors that develop in both C3(1)-SV40 Tag females and transplanted p53^-/- ^models have many features of "basal" tumors in women ([31,61], O'Leary and Schuler, in prep).

**Figure 4:**
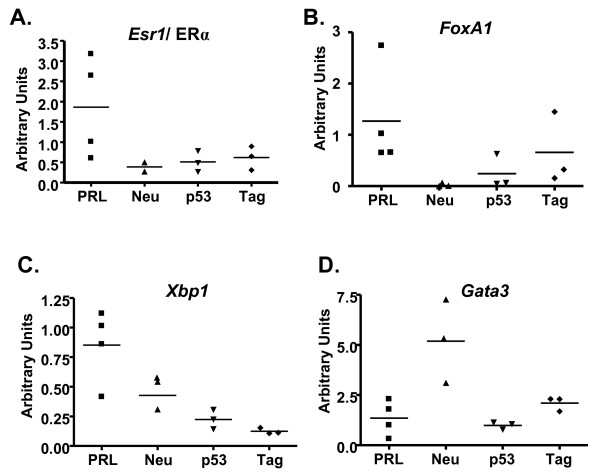
**PRL-induced adenocarcinomas display molecular features of luminal clinical breast tumors**. (**a-d**) RNA from mammary carcinomas that developed in NRL-PRL, MMTV-neu [28], C3(1)-SV40 Tag [29], and transplanted p53^-/- ^(O'Leary and Schuler, manuscript in preparation) models was examined for levels of transcripts comprising the luminal signature [31] by qRT-PCR as described in the Materials and Methods. PRL-induced papillary adenocarcinomas were of diverse ERα status by IHC. Each symbol denotes a distinct tumor. The horizontal bar indicates the mean level for each model.

Unlike the morphologically homogeneous MMTV-neu tumors, levels of transcripts that mark luminal breast cancers varied in NRL-PRL carcinomas, consistent with the diverse tumors in this model (Figure 4, Figure S1 in Additional File 1). In contrast to the majority of murine models, PRL-induced adenocarcinomas expressed relatively high levels of *Esr1 *mRNA, although as predicted from the IHC, levels varied considerably (Figure 4a). PRL-induced papillary carcinomas also contained comparatively high levels of the ERα-associated transcripts in the luminal signature, including *FoxA1 *and *Xbp1 *(Figure 4b, c). *Gata3 *mRNA was readily detectably in NRL-PRL tumors, but levels were lower than in MMTV-neu tumors (Figure 4d). Although Gata3 specifies ductal/alveolar cell fate and is markedly reduced in *Prlr*^-/- ^glands [1], this transcription factor also maintains luminal differentiation and is reduced in invasive breast cancer [62], consistent with the less differentiated phenotype of the PRL-induced carcinomas.

**Figure 5:**
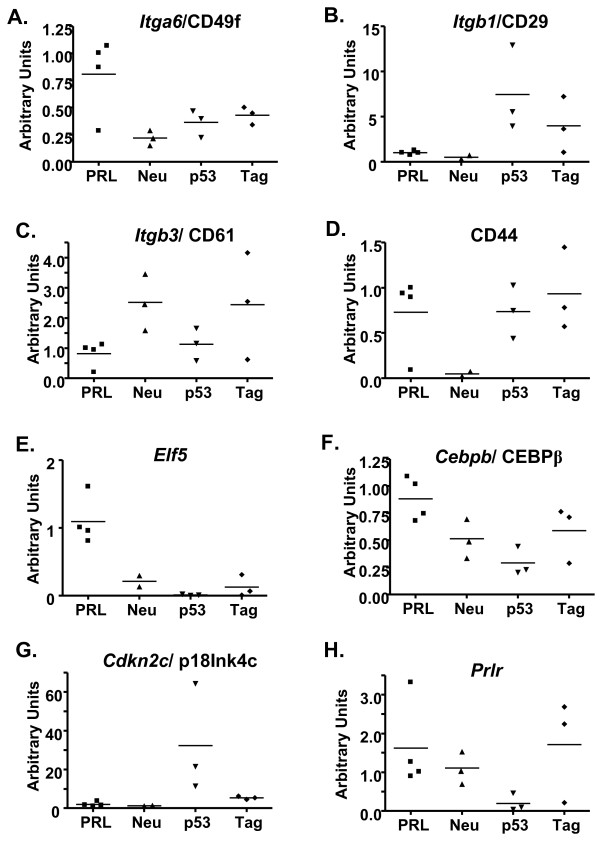
**NRL-PRL adenocarcinomas express markers distinct from other well-characterized models of breast cancer**. RNA from the set of mammary carcinomas in Figure 4 that developed in NRL-PRL, MMTV-neu [28], C3(1)-SV40 Tag [29], and transplanted p53^-/- ^(O'Leary and Schuler, manuscript in preparation) models was examined for levels of transcripts implicated in mammary lineages by qRT-PCR as described in the Materials and Methods. Each symbol denotes a distinct tumor. The horizontal bar indicates the mean level for each model.

In light of the luminal phenotype of many tumors promoted by PRL, we examined levels of transcripts associated with distinct mammary subpopulations and differentiation processes [24,63]. PRL-induced tumors displayed relatively high levels of *Itga6 *(encoding integrin α6, CD49f) mRNA, a marker for stem cell and progenitor populations (Figure 5a). They expressed relatively low levels of transcripts for both *Itgb1 *(encoding integrin β1, CD29), which marks stem cell and basal but not luminal subpopulations (Figure 5b), and also *Itgb3 *(encoding integrin β3, CD61), a marker for luminal progenitors (Figure 5c). In contrast, three of the four NRL-PRL adenocarcinomas displayed relatively high levels of CD44 (Figure 5d), an adhesion molecule associated with stem cells and metastasis [64,65]. NRL-PRL adenocarcinomas also contained elevated mRNA for several transcription factors, which direct luminal/alveolar cell expansion and differentiation, are co-expressed with PRLR in many normal MECs, and are linked to PRL in genetic models [1,66]. PRL-induced adenocarcinomas contained relatively high levels of mRNA for Elf5, a PRL-induced factor that directs the alveolar lineage [67], as well as CEBPβ, a transcriptional regulator which has been implicated in stem cell and luminal progenitor repopulation [68] (Figure 5e, f). Consistent with their roles in luminal cell commitment, individual genetic ablations of Gata3, Elf5, C/EBPβ and also Stat5 have been shown to alter the size of the MEC subpopulation expressing integrin β3 (CD61+), which is enriched in luminal precursors. However, the variable direction and timing of the observed changes reflect their complex actions at more than one stage of this process [24,68,69]. Future studies will elucidate the effect of PRL on mammary epithelial subpopulations and roles of these transcriptional regulators in its actions, and illuminate differences in cellular origin and/or pathways of progression from MMTV-neu tumors and among the different histotypes of PRL-induced carcinomas.

Transcripts for the cdk4/6 inhibitor, *Cdkn2c *(p18^INK4c^), were very low (Figure 5g). Genetic ablation of this tumor suppressor induces well-differentiated non-invasive luminal tumors in mouse models [70]. Similar results were observed for *Cdkn2a *(p16^INK4a^, data not shown). These results are consistent with the similar mammary phenotypes of *Prlr*^-/- ^and *Ccnd1*^-/- ^mice [1], and ability of PRL to increase cyclin D1 transcription [71], and support a key role for cdk4/6 in PRL-induced tumorigenesis.

PRL also may contribute to the tumor phenotype of other models; some C3(1)-SV40 Tag as well as MMTV-neu tumors contained appreciable *Prlr *mRNA (Figure 5h). The CD61+ progenitor population has recently been shown to be the origin of basal tumors [72]; the size of this MEC subpopulation is strongly modulated by Stat5. These observations invite speculation on potential therapeutic or preventative approaches directed at PRL action in multiple tumor types.

These marked differences in the molecular phenotype of PRL-induced tumors compared to other genetically modified mouse models of breast cancer suggest that PRL acts on its physiological target cells to promote diverse luminal-type tumors, but raises questions about the identity of these cells, the role of mediators associated with "normal" function in tumor progression, and factors contributing to tumor heterogeneity. Development of additional markers for the various mammary epithelial subpopulations, and antibodies that recognize the native conformation of the murine PRLR will assist in determining the characteristics of the precursors of luminal tumors, and identifying the target cells of PRL in this process.

### ERα expression is associated with distinct molecular features, but does not confer estrogen responsiveness

**Figure 6:**
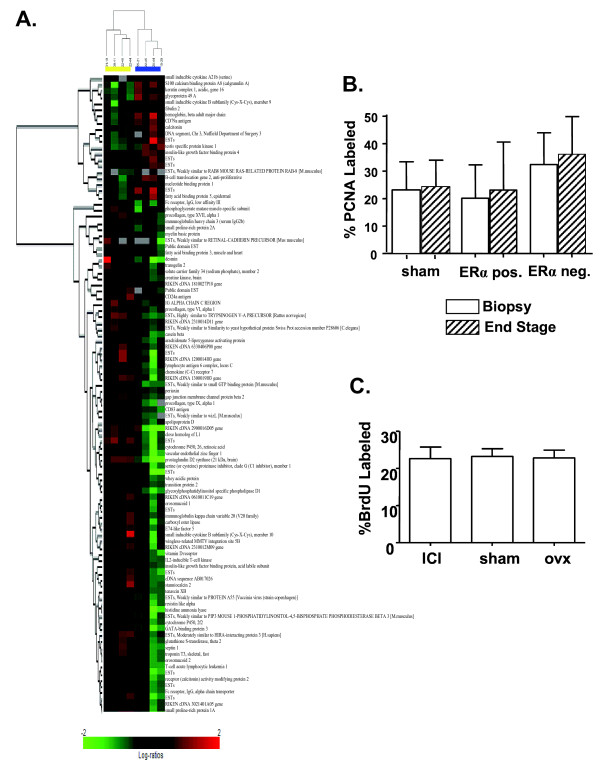
**ERα+ and ERα- carcinomas demonstrate distinct transcript profiles, and are insensitive to ovarian steroids**. **(a)** Hierarchical clustering of genes and tumor samples demonstrates differential expression between ERα+ (yellow) and ERα- (blue) adenocarcinomas at the *P *< 0.01 significance level (*N *= 4 each). ERα+ was defined as ≥10% of the cells contained detectable ERα by IHC; ERα- contained <10%. Red indicates high relative expression in ERα positive adenocarcinomas; green, low relative expression; black, no difference. Each column represents an independent tumor, and each row is a gene. **(b)** Growth of PRL-induced tumors is not diminished by ovariectomy, regardless of ERα status. Mammary carcinomas of distributed histotypes in NRL-PRL nonparous females were biopsied, and the mice subjected to sham surgery or ovariectomy. Subsequent tumor growth was monitored until tumors reached 1.5 cm in diameter (15.3 ± 8.5 days; mean ± s.d.), and proliferation determined by PCNA IHC as described in the Materials and Methods. Sham, *N *= 12; ovx ERα+, *N *= 10; ovx ERα-, *N *= 9. Treatment did not alter the rate of growth (Student's paired *t-*test, *P *> 0.05). **(c)** Treatment with the ER inhibitor and downregulator, Faslodex (ICI 182,780), does not inhibit growth of ERα positive tumor fragments. Fragments of well-differentiated ERα+ tumors were transplanted into fat pads of nontransgenic recipients and treated with ovariectomy, sham surgery, or Faslodex as described in Materials and Methods. Ovariectomized and Faslodex-treated females had significantly reduced uterine weights (27 ± 5 mg, 30 ± 8 mg, mean ± s.d., respectively), compared to sham-treated females (83 ± 5 mg). Treatment did not alter the rate of proliferation (*P *> 0.05).

In our preliminary studies comparing PRL-induced tumors to other murine models of breast cancer, PRL-induced ERα- and ERα+ tumors clustered together, indicating shared features suggesting a common origin. In order to determine characteristics of PRL-induced tumors that are associated with differences in ERα protein expression, we compared transcript profiles from four ERα+ and four ERα- adenocarcinomas (determined by IHC) of divergent histotypes that did not segregate with ERα status (three papillary, two glandular, one adenosquamous, two solid). Genes that were differentially expressed among the classes were determined, and hierarchical clustering analyses were performed by comparing expression profiles both across the set of samples and across the set of genes. As shown in Figure 6a, ERα+ and ERα- tumors clustered separately. Consistent with the reports of ERα+ tumors in women, NRL-PRL ERα+ tumors expressed higher levels of *Gata-3 *than ERα- tumors, and markers of differentiation, such as the milk proteins, β-casein and whey acidic protein, and CD24a [20,73-75]. Levels of mRNA for the PRL transgene and PRLR varied markedly among both ERα+ and ERα- carcinomas, suggesting that PRL activity itself was not responsible for the differences observed (Figure S2 in Additional File 1).

Activity of the NRL-PRL promoter is not altered by estrogen [15], enabling us to examine the role of this steroid in the disease process. Proliferation of both morphologically normal MECs and preneoplastic epithelial hyperplasias in NRL-PRL females is very sensitive to ovariectomy (ovx) and supplemental estrogen [17,54]. In order to examine estrogen responsiveness in established carcinomas, NRL-PRL females bearing primary mammary adenocarcinomas were subjected to sham surgery or ovx. At the time of surgery, a biopsy was removed for examination of tumor histology, rate of proliferation, and steroid hormone receptor expression. The growth of the remaining tumors was monitored until they reached 1.5 cm in diameter (15.3 ± 8.5 days; mean ± s.d.), as described in the Materials and methods. Like the tumors in Figure 1, histotypes in this experimental panel were diverse, and ERα/PR expression varied widely. They exhibited highly variable rates of growth (Figure 6b); rates of proliferation of ERα- tumors prior to the surgery tended to be higher than ERα+ tumors (*P *= 0.07). However, ovx did not alter rates of proliferation of either ERα+ or ERα- tumor cells. Consistently, ovx followed by administration of E2 failed to alter proliferation of an independent set of primary tumors (PCNA staining: 30.1% ± 12 vs 31.19% ± 7.9, mean ± s.d., *P *> 0.05, *N *= 5).

Since primary PRL-induced tumors exhibit highly variable histotype and ERα expression, and ovarian function is likely to be faltering in the aged females, we employed tumor transplantation to further investigate the role of ERα-dependent signals in established tumors. We serially passaged NRL-PRL glandular adenocarcinoma fragments that demonstrated high expression of both ERα and PR (48% and 25% labeled cells, respectively) to bilateral inguinal mammary glands of nonparous 12-week-old syngeneic nontransgenic recipients. Although the tumors that grew from these fragments (about eight weeks per passage) retained the morphology and high ERα expression of the original tumors, PR was no longer detectable after passage (not shown), suggesting that this process selects for more aggressive tumor cells. After three passages, recipient females were treated with ovx, sham surgery, or sham surgery and the ERα-selective antagonist, Faslodex. None of these treatments altered rates of proliferation of the tumors, as measured by BrdU incorporation (Figure 6c), indicating that relatively well-differentiated ERα+ tumors display the estrogen independence observed in the more diverse group of primary tumors.

These findings raise interesting questions regarding the mechanism(s) whereby PRL interacts with ERα-mediated signals in the development and progression of ERα+ tumors. The striking estrogen independence of established PRL-induced tumors shown herein contrasts with the cooperation between PRL and estrogen in proliferation of morphologically normal MECs and early lesions [17,54], suggesting acquisition of estrogen insensitivity. Many primary clinical ERα+ tumors acquire resistance to estrogen-directed therapies and most display marked therapeutic insensitivity after metastasis. A vast literature implicates many mechanisms, including aberrations not only of ERα itself, but alterations in other molecules and signaling pathways consistent with heightened growth factor activity [76-78]. The ability of PRL to potentiate growth factor signals [53-55,79] and high activation of AKT in the tumors shown here are consistent with a role in the latter mechanism. Clinically, high circulating PRL has been associated with failure of ERα+ tumors to respond to tamoxifen and aromatase inhibitors [80-82], and PRLR transcripts were elevated with recurrence after tamoxifen treatment in some studies [13,83]. The histological heterogeneity and variable activation of signaling pathways of PRL-induced tumors observed here suggest that multiple mechanisms may lead to loss of estrogen sensitivity. Together, these features suggest that the NRL-PRL model may provide insight into the origin of the diversity of clinical ERα tumors, and the characteristics of the tumor subpopulation that resists therapy.

## Conclusions

Despite the prevalence of ERα+ tumors clinically, very few mouse models resemble this breast cancer subtype. Our studies demonstrate that elevated local PRL can promote diverse carcinomas, which display varying levels of ERα/PR, nuclear pStat5, pERK1/2 and pAKT, and AP-1 components. Transcript analysis demonstrates that many of these carcinomas express the ERα-associated transcript signature that defines this clinical tumor subtype, and suggests that they arise from the physiologic target cells of PRL. In contrast to morphologically normal structures and hyperplastic lesions, established carcinomas are strikingly insensitive to estrogen, suggesting acquisition of independence. The role of ongoing PRL signals in established tumors will require additional investigation. Recent studies suggest that PRL also can antagonize traditional chemotherapeutics in breast cancer cells *in vitro *[84,85], which is supported by small studies of PRL on therapeutic resistance *in vivo *[86]. Together, our findings suggest that the NRL-PRL mammary tumor model will be helpful to understand the pathogenesis and heterogeneity of luminal tumors and identify factors determining susceptibility to anti-estrogen and traditional chemotherapies, to examine the role of PRL in these responses, and to test novel therapeutic strategies, including combinatorial regimens including therapies directed at PRL.

## Abbreviations

AP-1: Activating Protein 1; BrdU: bromodeoxyuridine; EMT: epithelial mesenchymal transition; ERα: estrogen receptor alpha; ERK: extracellular signal regulated kinase; IHC: immunohistochemistry; MECs: mammary epithelial cells; MMTV: mouse mammary tumor virus; PCNA: proliferating cell nuclear antigen; PR: progesterone receptor; PRL: prolactin; PRLR: prolactin receptor; qRT-PCR: quantitative reverse-transcriptase polymerase chain reaction; NRL: neu-related lipocalin; Stat5: signal transducer and activator of transcription 5.

## Competing interests

The authors declare that they have no competing interests.

## Authors' contributions

LMA and LAS contributed to concept design, data analysis and interpretation, and manuscript writing. DER carried out the transcript analyses and contributed to interpretation. TAG-H contributed to the generation of experimental data and analysis. MJG-B and HR analyzed the pStat5 expression of the tumors. All authors read and approved the final manuscript.

## Supplementary Material

Additional file 1 **Supplementary tables and figures**. Table S1: Immunohistochemistry conditions. Description of retrieval methods, blocking conditions and antibody dilutions used for each antigen examined. Table S2: Primers employed for RT-PCR analyses. Sequences of the primers used to quantify transcripts of interest. Figure S1: PRL-induced carcinomas of different histotypes display variable levels of transcripts for ERα-associated genes. Levels of mRNA for various transcripts of interest in individual PRL-induced tumors of different histotypes, determined by qRT-PCR. Figure S2: Levels of transgene and Prlr transcripts are variable in NRL-PRL adenocarcinomas, and are not associated with ERα status. Levels of mRNA for rPRL transgene and cytokeratin 8 (Krt8), and PRL receptor in individual tumors, determined by qRT-PCR. Click here for file

## References

[B1] Oakes SR Rogers RL Naylor MJ Ormandy CJ Prolactin regulation of mammary gland development J Mammary Gland Biol Neoplasia 2008 13 13 28 10.1007/s10911-008-9069-5 18219564

[B2] Tworoger SS Hankinson SE Prolactin and breast cancer etiology: an epidemiologic perspective J Mammary Gland Biol Neoplasia 2008 13 41 53 10.1007/s10911-008-9063-y 18246319

[B3] Bhatavdekar JM Patel DD Shah NG Vora HH Suthar TP Chikhlikar PR Ghosh N Trivedi TI Prognostic significance of immunohistochemically localized biomarkers in stage II and stage III breast cancer: a multivariate analysis Ann Surg Oncol 2000 7 305 311 10.1007/s10434-000-0305-5 10819372

[B4] Clevenger CV Zheng J Jablonski EM Galbaugh TL Fang F From bench to bedside: future potential for the translation of prolactin inhibitors as breast cancer therapeutics J Mammary Gland Biol Neoplasia 2008 13 147 156 10.1007/s10911-008-9074-8 18246318

[B5] McHale K Tomaszewski JE Puthiyaveettil R Livolsi VA Clevenger CV Altered expression of prolactin receptor-associated signaling proteins in human breast carcinoma Mod Pathol 2008 21 565 571 10.1038/modpathol.2008.7 18246042

[B6] Bhatavdekar JM Patel DD Shah NG Vora HH Suthar TP Ghosh N Chikhlikar PR Trivedi TI Prolactin as a local growth promoter in patients with breast cancer: GCRI experience Eur J Surg Oncol 2000 26 540 547 10.1053/ejso.2000.0943 11034803

[B7] Canbay E Degerli N Gulluoglu BM Kaya H Sen M Bardakci F Could prolactin receptor gene polymorphism play a role in pathogenesis of breast carcinoma? Curr Med Res Opin 2004 20 533 540 10.1185/030079904125003232 15119991

[B8] Vaclavicek A Hemminki K Bartram CR Wagner K Wappenschmidt B Meindl A Schmutzler RK Klaes R Untch M Burwinkel B Forsti A Association of prolactin and its receptor gene regions with familial breast cancer J Clin Endocrinol Metab 2006 91 1513 1519 10.1210/jc.2005-1899 16434456

[B9] Lee SA Haiman CA Burtt NP Pooler LC Cheng I Kolonel LN Pike MC Altshuler D Hirschhorn JN Henderson BE Stram DO A comprehensive analysis of common genetic variation in prolactin (PRL) and PRL receptor (PRLR) genes in relation to plasma prolactin levels and breast cancer risk: the multiethnic cohort BMC Med Genet 2007 8 72 10.1186/1471-2350-8-72 18053149 PMC2219987

[B10] Utama FE Tran TH Ryder A LeBaron MJ Parlow AF Rui H Insensitivity of human prolactin receptors to non-human prolactins: Relevance for experimental modeling of prolactin receptor-expressing human cells Endocrinology 2009 150 1782 1790 10.1210/en.2008-1057 19022890 PMC2659276

[B11] Perry JK Mohankumar KM Emerald BS Mertani HC Lobie PE The contribution of growth hormone to mammary neoplasia J Mammary Gland Biol Neoplasia 2008 13 131 145 10.1007/s10911-008-9070-z 18253708 PMC2665193

[B12] Swaminathan G Varghese B Fuchs SY Regulation of prolactin receptor levels and activity in breast cancer J Mammary Gland Biol Neoplasia 2008 13 81 91 10.1007/s10911-008-9068-6 18204982 PMC2276629

[B13] Rhodes DR Kalyana-Sundaram S Mahavisno V Varambally R Yu J Briggs BB Barrette TR Anstet MJ Kincead-Beal C Kulkarni P Varambally S Ghosh D Chinnaiyan AM Oncomine 3.0: genes, pathways, and networks in a collection of 18,000 cancer gene expression profiles Neoplasia 2007 9 166 180 10.1593/neo.07112 17356713 PMC1813932

[B14] Rose-Hellekant TA Arendt LM Schroeder MD Gilchrist K Sandgren EP Schuler LA Prolactin induces ERα-positive and ERα-negative mammary cancer in transgenic mice Oncogene 2003 22 4664 4674 10.1038/sj.onc.1206619 12879011 PMC1630768

[B15] Rose-Hellekant TA Schroeder MD Brockman JL Zhdankin O Bolstad R Chen KS Gould MN Schuler LA Sandgren EP Estrogen receptor positive mammary tumorigenesis in TGFα transgenic mice progresses with progesterone receptor loss Oncogene 2007 26 5238 5246 10.1038/sj.onc.1210340 17334393 PMC2587149

[B16] Arendt LM Schuler LA Transgenic models to study actions of prolactin in mammary neoplasia J Mammary Gland Biol Neoplasia 2008 13 29 40 10.1007/s10911-008-9073-9 18219562

[B17] Arendt LM Evans LC Rugowski DE Garcia-Barchino MJ Rui H Schuler LA Ovarian hormones are not required for PRL-induced mammary tumorigenesis, but estrogen enhances neoplastic processes J Endocrinol 2009 203 99 110 10.1677/JOE-09-0221 19635758 PMC2841967

[B18] Early Breast Cancer Trialists' Collaborative Group Effects of chemotherapy and hormonal therapy for early breast cancer on recurrence and 15-year survival: an overview of the randomised trials Lancet 2005 365 1687 1717 10.1016/S0140-6736(05)66544-0 15894097

[B19] Perou CM Borresen-Dale AL Systems biology and genomics of breast cancer Cold Spring Harb Perspect Biol 2010 in press 21047916 10.1101/cshperspect.a003293 PMC3039533

[B20] Hu Z Fan C Oh DS Marron JS He X Qaqish BF Livasy C Carey LA Reynolds E Dressler L Nobel A Parker J Ewend MG Sawyer LR Wu J Liu Y Nanda R Tretiakova M Ruiz OA Dreher D Palazzo JP Perreard L Nelson E Mone M Hansen H Mullins M Quackenbush JF Ellis MJ Olopade OI Bernard PS The molecular portraits of breast tumors are conserved across microarray platforms BMC Genomics 2006 7 96 10.1186/1471-2164-7-96 16643655 PMC1468408

[B21] Van de Vijver MJ He YD Van't Veer LJ Dai H Hart AA Voskuil DW Schreiber GJ Peterse JL Roberts C Marton MJ Parrish M Atsma D Witteveen A Glas A Delahaye L van d V Bartelink H Rodenhuis S Rutgers ET Friend SH Bernards R A gene-expression signature as a predictor of survival in breast cancer N Engl J Med 2002 347 1999 2009 10.1056/NEJMoa021967 12490681

[B22] Paik S Tang G Shak S Kim C Baker J Kim W Cronin M Baehner FL Watson D Bryant J Costantino JP Geyer CE Jr Wickerham DL Wolmark N Gene expression and benefit of chemotherapy in women with node-negative, estrogen receptor-positive breast cancer J Clin Oncol 2006 24 3717 3718 10.1200/JCO.2005.04.7985 16720680

[B23] Perreard L Fan C Quackenbush JF Mullins M Gauthier NP Nelson E Mone M Hansen H Buys SS Rasmussen K Orrico AR Dreher D Walters R Parker J Hu Z He X Palazzo JP Olopade OI Szabo A Perou CM Bernard PS Classification and risk stratification of invasive breast carcinomas using a real-time quantitative RT-PCR assay Breast Cancer Res 2006 8 R23 10.1186/bcr1399 16626501 PMC1557722

[B24] Visvader JE Keeping abreast of the mammary epithelial hierarchy and breast tumorigenesis Genes Dev 2009 23 2563 2577 10.1101/gad.1849509 19933147 PMC2779757

[B25] Prat A Perou CM Mammary development meets cancer genomics Nat Med 2009 15 842 844 10.1038/nm0809-842 19661985

[B26] Jacks T Remington L Williams BO Schmitt EM Halachmi S Bronson RT Weinberg RA Tumor spectrum analysis in p53-mutant mice Curr Biol 1994 4 1 7 10.1016/S0960-9822(00)00002-6 7922305

[B27] Hasen NS O'Leary KA Auger AP Schuler LA Social isolation reduces mammary development, tumor incidence and expression of epigenetic regulators in wild type and p53-heterozygotic mice Cancer Prev Res 2010 3 620 629 10.1158/1940-6207.CAPR-09-0225 PMC2865567 20424136

[B28] Guy CT Webster MA Schaller M Parsons TJ Cardiff RD Muller WJ Expression of the neu protooncogene in the mammary epithelium of transgenic mice induces metastatic disease Proc Natl Acad Sci USA 1992 89 10578 10582 10.1073/pnas.89.22.10578 1359541 PMC50384

[B29] Green JE Shibata MA Yoshidome K Liu ML Jorcyk C Anver MR Wigginton J Wiltrout R Shibata E Kaczmarczyk S Wang W Liu ZY Calvo A Couldrey C The C3(1)/SV40 T-antigen transgenic mouse model of mammary cancer: ductal epithelial cell targeting with multistage progression to carcinoma 2 Oncogene 2000 19 1020 1027 10.1038/sj.onc.1203280 10713685

[B30] Nevalainen MT Xie JW Bubendorf L Wagner KU Rui H Basal activation of transcription factor Stat5 in nonpregnant mouse and human breast epithelium Mol Endocrinol 2002 16 1108 1124 10.1210/me.16.5.1108 11981045

[B31] Herschkowitz JI Simin K Weigman VJ Mikaelian I Usary J Hu Z Rasmussen KE Jones LP Assefnia S Chandrasekharan S Backlund MG Yin Y Khramtsov AI Bastein R Quackenbush J Glazer RI Brown PH Green JE Kopelovich L Furth PA Palazzo JP Olopade OI Bernard PS Churchill GA van Dyke T Perou CM Identification of conserved gene expression features between murine mammary carcinoma models and human breast tumors Genome Biol 2007 8 R76 10.1186/gb-2007-8-5-r76 17493263 PMC1929138

[B32] Emerman JT Bissell MJ Cultures of mammary epithelial cells: extracellular matrix and functional differentiation Adv Cell Culture 1988 6 137 159

[B33] Gupta PB Proia D Cingoz O Weremowicz J Naber SP Weinberg RA Kuperwasser C Systemic stromal effects of estrogen promote the growth of estrogen receptor-negative cancers Cancer Res 2007 67 2062 2071 10.1158/0008-5472.CAN-06-3895 17332335

[B34] Wagner KU Rui H Jak2/Stat5 signaling in mammogenesis, breast cancer initiation and progression J Mammary Gland Biol Neoplasia 2008 13 93 103 10.1007/s10911-008-9062-z 18228120

[B35] Gutzman JH Rugowski DE Nikolai SE Schuler LA Stat5 activation inhibits prolactin-induced AP-1 activity: distinct prolactin initiated signals in tumorigenesis dependent on cell context Oncogene 2007 26 6341 6348 10.1038/sj.onc.1210454 17438530 PMC3190200

[B36] Gutzman JH Rugowski DE Schroeder MD Watters JJ Schuler LA Multiple kinase cascades mediate prolactin signals to activating protein-1 in breast cancer cells Mol Endocrinol 2004 18 3064 3075 10.1210/me.2004-0187 15319452 PMC1634796

[B37] Shaulian E Karin M AP-1 as a regulator of cell life and death Nat Cell Biol 2002 4 E131 E136 10.1038/ncb0502-e131 11988758

[B38] Eferl R Wagner EF AP-1: a double-edged sword in tumorigenesis Nature Reviews Cancer 2003 3 859 868 10.1038/nrc1209 14668816

[B39] Ozanne BW Spence HJ McGarry LC Hennigan RF Transcription factors control invasion: AP-1 the first among equals Oncogene 2007 26 1 10 10.1038/sj.onc.1209759 16799638

[B40] Robinson GW McKnight RA Smith GH Hennighausen L Mammary epithelial cells undergo secretory differentiation in cycling virgins but require pregnancy for the establishment of terminal differentiation Development 1995 121 2079 2090 7635053 10.1242/dev.121.7.2079

[B41] Harris J Stanford PM Sutherland K Oakes SR Naylor MJ Robertson FG Blazek KD Kazlauskas M Hilton HN Wittlin S Alexander WS Lindeman GJ Visvader JE Ormandy CJ Socs2 and Elf5 mediate prolactin-induced mammary gland development Mol Endocrinol 2006 20 1177 1187 10.1210/me.2005-0473 16469767

[B42] Wu ZS Wu Q Yang JH Wang HQ Ding XD Yang F Xu XC Prognostic significance of MMP-9 and TIMP-1 serum and tissue expression in breast cancer Int J Cancer 2008 122 2050 2056 10.1002/ijc.23337 18172859

[B43] Whyte J Bergin O Bianchi A McNally S Martin F Key signalling nodes in mammary gland development and cancer. Mitogen-activated protein kinase signalling in experimental models of breast cancer progression and in mammary gland development Breast Cancer Res 2009 11 209 10.1186/bcr2361 19818165 PMC2790844

[B44] Thiery JP Sleeman JP Complex networks orchestrate epithelial-mesenchymal transitions Nat Rev Mol Cell Biol 2006 7 131 142 10.1038/nrm1835 16493418

[B45] Turley EA Veiseh M Radisky DC Bissell MJ Mechanisms of disease: epithelial-mesenchymal transition--does cellular plasticity fuel neoplastic progression? Nat Clin Pract Oncol 2008 5 280 290 10.1038/ncponc1089 18349857 PMC2846172

[B46] Dillon RL White DE Muller WJ The phosphatidyl inositol 3-kinase signaling network: implications for human breast cancer Oncogene 2007 26 1338 1345 10.1038/sj.onc.1210202 17322919

[B47] Tokunaga E Kataoka A Kimura Y Oki E Mashino K Nishida K Koga T Morita M Kakeji Y Baba H Ohno S Maehara Y The association between Akt activation and resistance to hormone therapy in metastatic breast cancer Eur J Cancer 2006 42 629 635 10.1016/j.ejca.2005.11.025 16464571

[B48] Kirkegaard T Witton CJ McGlynn LM Tovey SM Dunne B Lyon A Bartlett JM AKT activation predicts outcome in breast cancer patients treated with tamoxifen J Pathol 2005 207 139 146 10.1002/path.1829 16088978

[B49] Sakamoto K Triplett AA Schuler LA Wagner KU Jak2 is required for the initiation but not maintenance of prolactin-induced mammary cancer Oncogene 2010 29 5359 5369 10.1038/onc.2010.274 20639901 PMC2997721

[B50] Schiff R Reddy P Ahotupa M Coronado-Heinsohn E Grim M Hilsenbeck SG Lawrence R Deneke S Herrera R Chamness GC Fuqua SA Brown PH Osborne CK Oxidative stress and AP-1 activity in tamoxifen-resistant breast tumors *in vivo* J Natl Cancer Inst 2000 92 1926 1934 10.1093/jnci/92.23.1926 11106684

[B51] Vendrell JA Robertson KE Ravel P Bray SE Bajard A Purdie CA Nguyen C Hadad SM Bieche I Chabaud S Bachelot T Thompson AM Cohen PA A candidate molecular signature associated with tamoxifen failure in primary breast cancer Breast Cancer Res 2008 10 R88 10.1186/bcr2158 18928543 PMC2614524

[B52] Creighton CJ Kent OC Van de Vijver MJ Foekens JA Klijn JG Horlings HM Nuyten D Wang Y Zhang Y Chamness GC Hilsenbeck SG Lee AV Schiff R Molecular profiles of progesterone receptor loss in human breast tumors Breast Cancer Res Treat 2008 114 287 299 10.1007/s10549-008-0017-2 18425577 PMC2635926

[B53] Arendt LM Rose-Hellekant TA Sandgren EP Schuler LA Prolactin potentiates TGFα induction of mammary neoplasia in transgenic mice Am J Pathol 2006 168 1365 1374 10.2353/ajpath.2006.050861 16565509 PMC1606572

[B54] Arendt LM Grafwallner-Huseth TL Schuler LA Prolactin-growth factor crosstalk reduces mammary estrogen responsiveness despite elevated ERα expression Am J Pathol 2009 174 1065 1074 10.2353/ajpath.2009.080719 19179608 PMC2665765

[B55] Carver KC Schuler LA Prolactin does not require insulin-like growth factor (IGF) intermediates, but synergizes with IGF-1 in human breast cancer cells Mol Cancer Res 2008 6 634 643 10.1158/1541-7786.MCR-07-2069 18403642

[B56] Carroll JS Liu XS Brodsky AS Li W Meyer CA Szary AJ Eeckhoute J Shao W Hestermann EV Geistlinger TR Fox EA Silver PA Brown M Chromosome-wide mapping of estrogen receptor binding reveals long-range regulation requiring the forkhead protein FoxA1 Cell 2005 122 33 43 10.1016/j.cell.2005.05.008 16009131

[B57] Bernardo GM Lozada KL Miedler JD Harburg G Hewitt SC Mosley JD Godwin AK Korach KS Visvader JE Kaestner KH Abdul-Karim FW Montano MM Keri RA FOXA1 is an essential determinant of ERalpha expression and mammary ductal morphogenesis Development 2010 137 2045 2054 10.1242/dev.043299 20501593 PMC2875844

[B58] Ursini-Siegel J Schade B Cardiff RD Muller WJ Insights from transgenic mouse models of ERBB2-induced breast cancer Nat Rev Cancer 2007 7 389 397 10.1038/nrc2127 17446858

[B59] Vaillant F Asselin-Labat ML Shackleton M Forrest NC Lindeman GJ Visvader JE The mammary progenitor marker CD61/beta3 integrin identifies cancer stem cells in mouse models of mammary tumorigenesis Cancer Res 2008 68 7711 7717 10.1158/0008-5472.CAN-08-1949 18829523

[B60] Henry MD Triplett AA Oh KB Smith GH Wagner KU Parity-induced mammary epithelial cells facilitate tumorigenesis in MMTV-neu transgenic mice Oncogene 2004 23 6980 6985 10.1038/sj.onc.1207827 15286714

[B61] Deeb KK Michalowska AM Yoon CY Krummey SM Hoenerhoff MJ Kavanaugh C Li MC DeMayo FJ Linnoila I Deng CX Lee EY Medina D Shih JH Green JE Identification of an integrated SV40 T/t-antigen cancer signature in aggressive human breast, prostate, and lung carcinomas with poor prognosis Cancer Res 2007 67 8065 8080 10.1158/0008-5472.CAN-07-1515 17804718

[B62] Kouros-Mehr H Kim JW Bechis SK Werb Z GATA-3 and the regulation of the mammary luminal cell fate Curr Opin Cell Biol 2008 20 164 170 10.1016/j.ceb.2008.02.003 18358709 PMC2397451

[B63] Stingl J Detection and analysis of mammary gland stem cells J Pathol 2009 217 229 241 10.1002/path.2457 19009588

[B64] Godar S Ince TA Bell GW Feldser D Donaher JL Bergh J Liu A Miu K Watnick RS Reinhardt F McAllister SS Jacks T Weinberg RA Growth-inhibitory and tumor- suppressive functions of p53 depend on its repression of CD44 expression Cell 2008 134 62 73 10.1016/j.cell.2008.06.006 18614011 PMC3222460

[B65] Wang Y Klijn JG Zhang Y Sieuwerts AM Look MP Yang F Talantov D Timmermans M Meijer-van Gelder ME Yu J Jatkoe T Berns EM Atkins D Foekens JA Gene-expression profiles to predict distant metastasis of lymph-node-negative primary breast cancer Lancet 2005 365 671 679 15721472 10.1016/S0140-6736(05)17947-1

[B66] Grimm SL Seagroves TN Kabotyanski EB Hovey RC Vonderhaar BK Lydon JP Miyoshi K Hennighausen L Ormandy CJ Lee AV Stull MA Wood TL Rosen JM Disruption of steroid and prolactin receptor patterning in the mammary gland correlates with a block in lobuloalveolar development Mol Endocrinol 2002 16 2675 2691 10.1210/me.2002-0239 12456789

[B67] Oakes SR Naylor MJ Asselin-Labat ML Blazek KD Gardiner-Garden M Hilton HN Kazlauskas M Pritchard MA Chodosh LA Pfeffer PL Lindeman GJ Visvader JE Ormandy CJ The Ets transcription factor Elf5 specifies mammary alveolar cell fate Genes Dev 2008 22 581 586 10.1101/gad.1614608 18316476 PMC2259028

[B68] LaMarca HL Visbal AP Creighton CJ Liu H Zhang Y Behbod F Rosen JM C/EBPbeta regulates stem cell activity and specifies luminal cell fate in the mammary gland Stem Cells 2010 28 535 544 20054865 10.1002/stem.297 PMC3006225

[B69] Yamaji D Na R Feuermann Y Pechhold S Chen W Robinson GW Hennighausen L Development of mammary luminal progenitor cells is controlled by the transcription factor STAT5A Genes Dev 2009 23 2382 2387 10.1101/gad.1840109 19833766 PMC2764497

[B70] Pei XH Bai F Smith MD Usary J Fan C Pai SY Ho IC Perou CM Xiong Y CDK inhibitor p18(INK4c) is a downstream target of GATA3 and restrains mammary luminal progenitor cell proliferation and tumorigenesis Cancer Cell 2009 15 389 401 10.1016/j.ccr.2009.03.004 19411068 PMC2699569

[B71] Brockman JL Schroeder MD Schuler LA Prolactin activates the cyclin D1 promoter via the JAK2-STAT pathway Mol Endocrinol 2002 16 774 784 10.1210/me.16.4.774 11923474

[B72] Lim E Vaillant F Wu D Forrest NC Pal B Hart AH Asselin-Labat ML Gyorki DE Ward T Partanen A Feleppa F Huschtscha LI Thorne HJ Fox SB Yan M French JD Brown MA Smyth GK Visvader JE Lindeman GJ Aberrant luminal progenitors as the candidate target population for basal tumor development in BRCA1 mutation carriers Nat Med 2009 15 907 913 10.1038/nm.2000 19648928

[B73] Gruvberger S Ringnér M Chen YD Panavally S Saal LH Borg Å Fernö M Peterson C Meltzer PS Estrogen receptor status in breast cancer is associated with remarkably distinct gene expression patterns Cancer Res 2001 61 5979 5984 11507038

[B74] Van't Veer LJ Dai HY Van de Vijver MJ He YDD Hart AAM Mao M Peterse HL Van der Kooy K Marton MJ Witteveen AT Schreiber GJ Kerkhoven RM Roberts C Linsley PS Bernards R Friend SH Gene expression profiling predicts clinical outcome of breast cancer Nature 2002 415 530 536 11823860 10.1038/415530a

[B75] Sorlie T Molecular portraits of breast cancer: tumour subtypes as distinct disease entities Eur J Cancer [A] 2004 40 2667 2675 10.1016/j.ejca.2004.08.021 15571950

[B76] Ali S Coombes RC Endocrine-responsive breast cancer and strategies for combating resistance Nat Rev Cancer 2002 2 101 112 10.1038/nrc721 12635173

[B77] Musgrove EA Sutherland RL Biological determinants of endocrine resistance in breast cancer Nat Rev Cancer 2009 9 631 643 10.1038/nrc2713 19701242

[B78] Nicholson RI Hutcheson IR Jones HE Hiscox SE Giles M Taylor KM Gee JM Growth factor signalling in endocrine and anti-growth factor resistant breast cancer Rev Endocr Metab Disord 2007 8 241 253 17486454 10.1007/s11154-007-9033-5

[B79] Carver KC Arendt LM Schuler LA Complex prolactin crosstalk in breast cancer: new therapeutic implications Mol Cell Endocrinol 2009 307 1 7 10.1016/j.mce.2009.03.014 19524120 PMC3190192

[B80] Barni S Lissoni P Meregalli S Ardizzoia A Mengo S Musco F Merlini D Tancini G Clinical efficacy of the aromatase inhibitor anastrozole in relation to prolactin secretion in heavily pretreated metastatic breast cancer Tumori 1998 84 45 47 9619713 10.1177/030089169808400109

[B81] Bhatavdekar JM Patel DD Karelia NH Shah NG Ghosh N Vora HH Suthar TP Balar DB Doctor SS Can plasma prolactin predict tamoxifen resistance in patients with advanced breast cancer? Eur J Surg Oncol 1994 20 118 121 8181575

[B82] Dowsett M McGarrick GE Harris AL Coombes RC Smith IE Jeffcoate SL Prognostic significance of serum prolactin levels in advanced breast cancer Br J Cancer 1983 47 763 769 10.1038/bjc.1983.129 6860546 PMC2011370

[B83] Ma XJ Salunga R Tuggle JT Gaudet J Enright E McQuary P Payette T Pistone M Stecker K Zhang BM Zhou YX Varnholt H Smith B Gadd M Chatfield E Kessler J Baer TM Erlander MG Sgroi DC Gene expression profiles of human breast cancer progression Proc Natl Acad Sci USA 2003 100 5974 5979 10.1073/pnas.0931261100 12714683 PMC156311

[B84] LaPensee EW Schwemberger SJ LaPensee CR Bahassi EM Afton S Ben-Jonathan N Prolactin confers resistance against cisplatin in breast cancer cells by activating glutathione-S-transferase Carcinogenesis 2009 30 1298 1304 10.1093/carcin/bgp120 19443905 PMC2718070

[B85] Howell SJ Anderson E Hunter T Farnie G Clarke RB Prolactin receptor antagonism reduces the clonogenic capacity of breast cancer cells and potentiates doxorubicin and paclitaxel cytotoxicity Breast Cancer Res 2008 10 R68. 18681966 10.1186/bcr2129 PMC2575541

[B86] Frontini L Lissoni P Vaghi M Perego MS Pescia S Ardizzoia A Gardani G Enhancement of the efficacy of weekly low-dose taxotere by the long acting anti-prolactinemic drug cabergoline in pretreated metastatic breast cancer Anticancer Res 2004 24 4223 4226 15736476

