# Supplementary material for: Prolactin-induced mouse mammary carcinomas model estrogen resistant luminal breast cancer
Source: Breast Cancer Res. 2011 Jan 28;13(1):R11. doi: 10.1186/bcr2819 (PMC3109579; doi:10.1186/bcr2819)
Supplement: Additional file 1 — Supplementary tables and figures. Table S1: Immunohistochemistry conditions. Description of retrieval methods, blocking conditions and antibody dilutions used for each antigen examined. Table S2: Primers employed for RT-PCR analyses. Sequences of the primers used to quantify transcripts of interest. Figure S1: PRL-induced carcinomas of different histotypes display variable levels of transcripts for ERα-associated genes. Levels of mRNA for various transcripts of interest in individual PRL-induced tumors of different histotypes, determined by qRT-PCR. Figure S2: Levels of transgene and Prlr transcripts are variable in NRL-PRL adenocarcinomas, and are not associated with ERα status. Levels of mRNA for rPRL transgene and cytokeratin 8 (Krt8), and PRL receptor in individual tumors, determined by qRT-PCR. [file bcr2819-S1.PDF]

Table S1. Immunohistochemistry conditions

	Antigen Retrieval	Block	Dilution
PCNA	citrate	M.O.M. diluent	1:400
PR	citrate	5% BSA/TBST	1:750
ERα	tris	1% rabbit serum/TBST	1:1000
BrdU	tris	1% milk/PBS	1:40
P-ERK 1/2	tris	1% rabbit serum/TBST	1:100
P-Stat 5	citrate	3% horse serum/PBS	1:750
c-Fos	tris	1% milk/PBS	1:500
c-Jun	citrate	5% goat serum/PBS	1:250
P-Akt	tris	1% milk/PBS	1:50

Table S2. Primers employed for RT-PCR analyses

Cdkn2C Ta=60C
Forward 5’ CAA CGC CCC GAA CTC TTT C 3’
Reverse 5’ AGC AGA AGA GCT GCT ACG TGA A 3’

CD44 Ta=60C
Forward 5’ CCA GGC TTT CAA CAG TAC CTT ACC 3’
Reverse 5’ TGA ACC CAT ACC TGC ATG TTT C 3’

Cebpb Ta=60C
Forward 5’ AAG CTG AGC GAC GAG TAC AAG A 3’
Reverse 5’ GTC AGC TCC AGC ACC TTG TG 3’

Elf5 Ta=60C
Forward 5’ ATG CCT TGG ACC GAT CTG TT 3’
Reverse 5’ GTG CAC TGA TGT CCA GTA GGA ATC 3’

ERα Ta=60C
Forward 5’ GCC AGA ATG GCC GAG AGA 3’
Reverse 5’ TCA TTG CAC ACG GCA CAG T 3’

Expi Ta=60C
Forward 5’ GGG CTC TGT CTA ACC CCA AAG 3’
Reverse 5’ GCA TCG TTC ATC ACA AGT TCC A 3’

Gata3 Ta=60C
Forward 5’ CGA GAT GGT ACC GGG CAC TA 3’
Reverse 5’ GAC AGT TCG CGC AGG ATG T 3’

Itga6 Ta=60C
Forward 5’ GAT GGG CCC TAT GAA GTT GGT 3’
Reverse 5’ CCC TTC CCT GAG TCC AGT GA 3’

Itgb1 Ta=60C
Forward 5’ GGT GGC TTT GAT GCA ATC ATG 3’
Reverse 5’ CCA GCA TCC GTG GAA AAC AC 3’

Itgb3 Ta=58C
Forward 5’ GGC AAT CAA AAA CCC CTG TTA C 3’
Reverse 5’ TTA GCG TCA GCA CGT GTT TGT 3’

Keratin 19 Ta=60C
Forward 5’ CCA AGA TCC TGA GTG AGA TGA GAA 3’
Reverse 5’ TCG GTC TTG CTT ATC TGG ATC TG 3’

Keratin 8 Ta=60C
Forward 5’ TGA ACA ACA AGT TCG CCT CCT T 3’
Reverse 5’ TCC ACT TGG TCT CCA GCA TCT 3’

MMP9 Ta=60C
Forward 5’ TGG TGT GCC CTG GAA CTC A 3’
Reverse 5’ AGG TCG TAG GTC ACG TAG CCC A 3’

PRLR Ta=60
Forward 5’ TCT CTC TGC TGT CAT CTG TTT GAT T 3’
Reverse 5’ GAA AGA TGC AGG TCA TCA TGA TAT 3’

Vimentin Ta=60C
Forward 5’ CGG AAA GAA TCC TTG CA 3’
Reverse 5’ GGA AAC GTC CAC ATC GAT CTG 3’

Xbp1 Ta=60C
Forward 5’ CAG CAA GTG GTG GAT TTG GA 3’
Reverse 5’ CGT GTT CTT AAC TCC TGG TTC TCA 3’

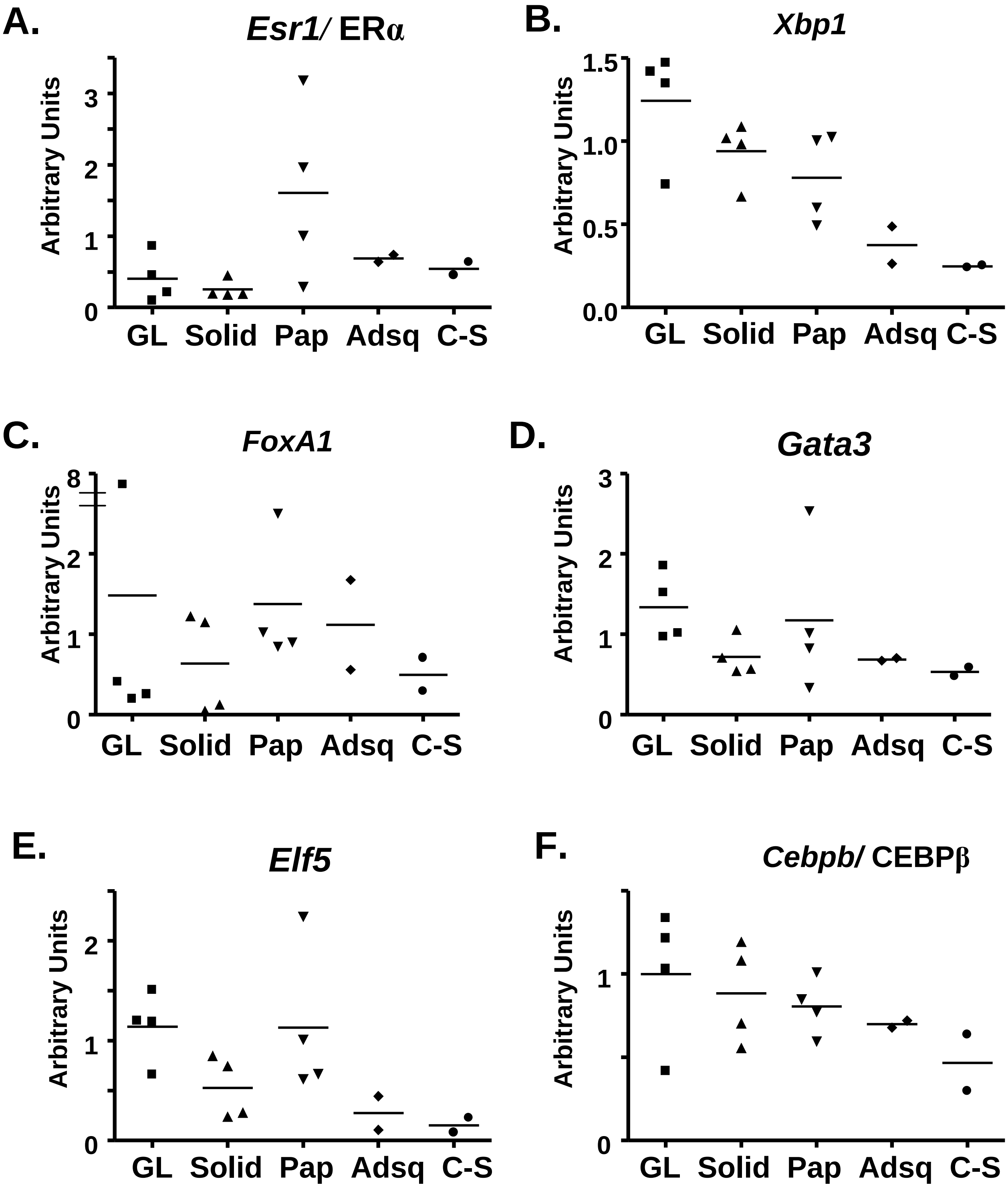

Fig. S1. PRL-induced carcinomas of different histotypes display variable levels of transcripts for ERα-associated genes. RNA from ERα+ and ERα- carcinomas (Table 1) was examined for relative levels of the transcripts shown by qRT-PCR as described in the Methods. Each individual symbol represents a single tumor.

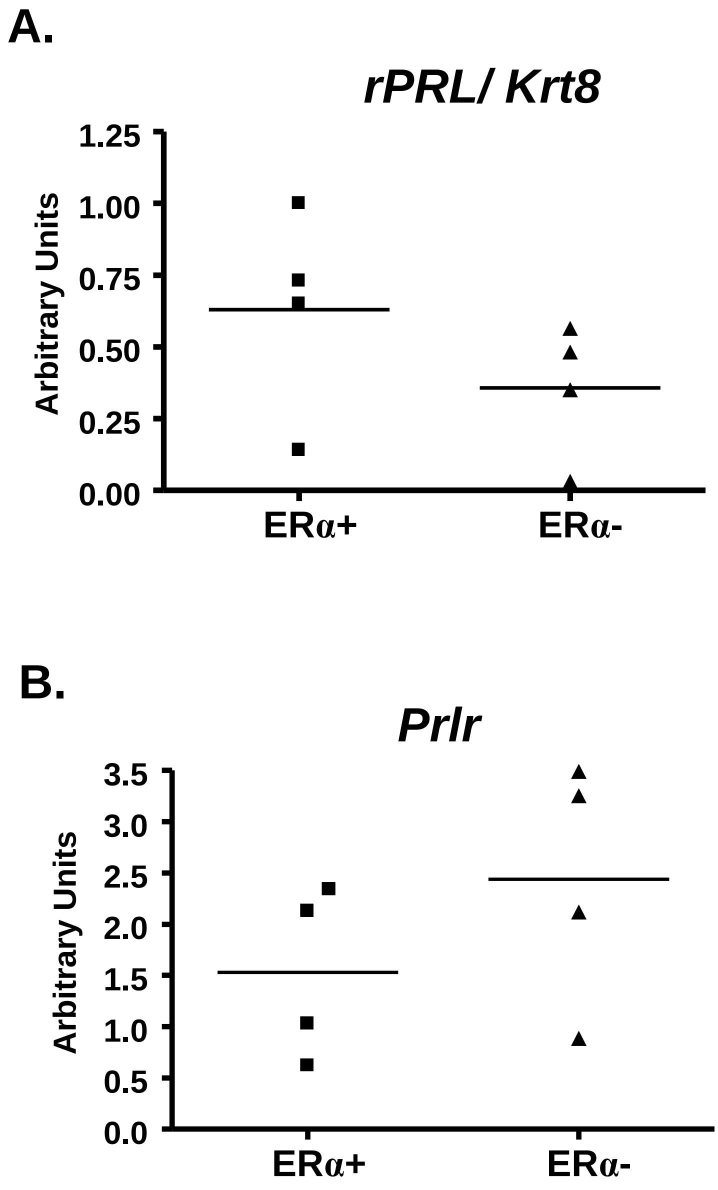

Fig S2. Levels of transgene and Prlr transcripts are variable in NRL-PRL adenocarcinomas, and are not associated with ERα status. RNA from the tumors profiled in Fig. 6A was examined for (A) relative levels of the rPRL transgene and cytokeratin 8 (Krt8), and (B) PRL receptor (Prlr), by qRT-PCR as described in the Methods. Each individual symbol represents a single tumor.
